# New Insights Into the Effects of Individual Chinese Herbal Medicines on Chronic Kidney Disease

**DOI:** 10.3389/fphar.2021.774414

**Published:** 2021-11-04

**Authors:** Minghai Shao, Chaoyang Ye, George Bayliss, Shougang Zhuang

**Affiliations:** ^1^ Department of Nephrology, Shuguang Hospital, Shanghai University of Traditional Chinese Medicine, Shanghai, China; ^2^ Department of Medicine, Rhode Island Hospital and Alpert Medical School, Brown University, Providence, RI, United States; ^3^ Department of Nephrology, Shanghai East Hospital, Tongji University School of Medicine, Shanghai, China

**Keywords:** renal fibrosis, Chinese herbs, monomers, chronic kidney disease, *Abelmoschus manihot*, *Salvia miltiorrhiza*, therapeutic molecular mechanism

## Abstract

The clinical and experimental study into the effects of Chinese herbal medicines on chronic kidney disease has evolved over the past 40 years with new insight into their mechanism and evidence of their clinical effects. Among the many traditional Chinese herbs examined in chronic renal disease, five were found to have evidence of sufficient clinical efficacy, high frequency of use, and well-studied mechanism. They are: *Abelmoschus manihot* and *Huangkui capsule*, *Salvia miltiorrhiza* and its components (tanshinone II A, salvianolic acid A and B); *Rhizoma coptidis* and its monomer *berberine*; *Tripterygium wilfordii* and its components (triptolide, tripterygium glycosides); Kudzu root *Pueraria* and its monomer *Puerarin*. These Chinese herbal medications have pharmaceutical effects against fibrosis, inflammation and oxidative stress and also promote renal repair and regeneration. This article reviews their clinical efficacy, anti-fibrotic effects in animal models, and molecular mechanism of action.

## Introduction

Chronic kidney disease (CKD) is a global public health issue, affecting more than 10% of the world’s population (Glassock et al., 2017; Ruiz-Ortega et al., 2020). The burden of CKD is not only restricted to the requirement of renal replacement therapy for end stage of renal disease (ESRD), but also associated with cardiovascular events and mortality (Glassock et al., 2017). Although the etiology of CKD and pathological course are diverse, renal interstitial fibrosis and gradual loss of nephron mass are the common pathological changes. Renal fibrosis is characterized by activation of renal interstitial fibroblasts and deposition of extracellular matrix components that are driven by multiple signaling pathways, transcriptional factors, inflammatory factors, oxidative stress and vasoactive substances, including angiotensin (Liu 2011; Meng, et al., 2016; Ruiz-Ortega, et al., 2020). Current treatment of patients with CKD most still relies mostly on an angiotensin-converting enzyme inhibitor (ACEI) and an angiotensin receptor blocker (ARB), however, these drugs only ameliorate, but not halt the progression of CKD to ESRD (Ruiz-Ortega et al., 2020). The limitations of Western medicine in curing or slowing progression of CKD may drive some patients to seek alternative treatments such as Chinese herbal medicines.

Chinese herbal medicines have been extensively used to treat CKD and other chronic diseases in China and some Asian countries. However, high-quality clinical evidence is lacking to support use of Chinese herbal medicines for CKD treatment worldwide. In 2015, Lin et al., published the first population-based retrospective cohort study on the use of Chinese herbal medicine in CKD patients (Lin et al., 2015). By searching for the Taiwan National Health Insurance Research Database from 2000 to 2005, they found that among the 24,971 study patients, 11,351 received prescribed Chinese herbal medicine after CKD diagnosis. After adjusting confounding variable, the group using Chinese herbal medicine exhibited a significantly reduced ESRD risk (60%) compared with the nonuse group. This provides solid evidence of the association between the use of Chinese herbal medicines with reduced ESRD risk in patients with CKD. Further analysis of Chinese herbal medicines used in this population of CKD patients revealed that the formulas classified as “blood-regulating,” “dampness-dispelling,” or “harmonizing” were strongly associated with the protection effect against CKD (Lin et al., 2015), suggesting that these classes of Chinese herbal formulas contain therapeutic components that prevent CKD progression.

A Chinese herbal medicine formula usually contains several medicinal herbs. Identifying the role of individual herbs is essential for understanding the role and mechanism of a Chinese herbal medicine formula in treating various diseases including CKD. In the past 40 years, many such studies have been conducted to search for medicinal herbs that are effective for treating CKD. Five were found to have evidence of sufficient clinical efficacy, high frequency of use, and well-studied mechanism. These herbs include *Abelmoschus Manihot*, *Salvia miltiorrhiza, Rhizoma coptidis*, *Tripterygium wilfordii*, and *Kudzu root Pueraria.* Interestingly, these five Chinese medicinal herbs are also major components in either blood-regulating, dampness-dispelling, heat-clearing, or harmonizing formula associated with beneficial effect to CKD patients as mentioned above (Lin et al., 2015). Moreover, the extract and/or monomer of these five medicinal herbs have been made and tested in animal models of CKD and/or patents with CKD (see below). A prospective, open-label, multicenter, randomized controlled trial demonstrated that Huangkui capsule, a single-plant drug extracted from the dry corolla of Flos A. Manihot, was more effective than the angiotensin-receptor blocker losartan in reducing proteinuria in patients with primary glomerular disease after 24 weeks of treatment (Zhang, et al., 2014), which resulted in its approval by the China Food and Drug Administration to treat CKD stages 1–2 with primary glomerular disease.

In this article, we review the therapeutic effect of these five medicinal herbs in animal models of CKD (Table 1) and their clinical efficacy in CKD patients (Table 2) as well as molecular mechanism of their actions. We also discuss the challenge and directions of medicinal herb research associated with CKD.

**TABLE 1:** Recent animal studies on Chinese herbal medicines with anti-renal fibrosis function.

Herbal/extract	Animal model	Outcome	Mechanism	Reference
Abelmoschus Manihot	5/6 nephrectomy	↓EMT	↓PI3K-Akt-eNOS, ERK1/2	Gu, et al. (2020); Peng, et al. (2016)
	ADRN	↓OX, inflammation	↓ROS-ERK1/2-NLRP3	Li, et al. (2019); Cai, et al. (2017)
			↓NADPH oxidase/ROS/ERK	
	UNE-ADR	↓inflammation, glomerulosclerosis	↓TNF-α,TGF-β1, p38MAPK	Tu, et al. (2013)
	STZ-DN mice	↓OX	↑AMPK-Sirt1-PGC-1	Liao, et al. (2019)
	UNE-STZ-DN	↓podocyte apoptosis	↑PPAR-α/γ	Ge, et al. (2016); Liu, et al. (2017)
			↓ iRhom2/TACE,ERS	
	UNE-STZ-HFD-DN	↓podocyte loss, FN	Regulating autophagy, mitochondrial dynamics	Kim, et al. (2018)
	UPPR rat	↑intestinal microbiota	autophagy-mediated macrophage polarization	Tu, et al. (2020)
		↓micro-inflammation		
Salvianolic Acid A, Tanshinone IIA	5/6 nephrectomy	↓OX, inflammation	↑Akt/GSK-3β/Nrf2, BMP-7, Smad6	Zhang, et al. (2019b); Zhang, et al. (2019a); Zhang, et al. (2018); Wang, et al. (2015b)
			↓NF-kB, p38 MAPK, TGF-β/Smads	
Salvianolic acid A	ADR-MCD rats	↓proteinuria, podocyte injury	↑PPAR-γ/Angptl4, Nrf2/HO-1	Wang, et al. (2019b)
Tanshinone IIA	STZ-DN	↓ERS, albuminuria	↓PERK	(Xu, et al., 2020a)
		↓pathological damage	↓OX, inflammation	Chen, et al. (2017)
Salvianolic Acid A	STZ-HFD-DN	↓OX, inflammation, endothelial permeability; ↑autophagy	↓AGE-RAGE-RhoA/ROCK, AGE-RAGE-Nox4 axis	Hou, et al. (2017)
Salvianolic Acid B	UUO rat	↓pathological damage	↓heparanase/syndecan 1	Hu, et al. (2020)
Salvianolic Acid A, C	UUO rat	↑renal function, tubular function	↓CCL5 and CXCL10	Li, et al. (2015)
		↓pathological damage		
Tanshinone IIA	AD-PO-UAN	↓OX	↓ NOX4, MAPK	Zhang et al. (2020c)
Salvianolic Acid B	RIRI rats	↓OX, inflammation; caspase-1-mediated pyroptosis	↑ PI3K/Akt; ↓ Nrf2 pathway	Ma et al., (2017b)
				Pang, et al. (2020)
Tanshinone IIA	ioversol-CIN	↓tubular necrosis, apoptosis, OX	↑ Nrf2/ARE activation	Liang, et al. (2018)
Tanshinone I	AAI-KI	↓kidney injury	↑cytochrome P450 1A	Feng, et al. (2013)
Salvianolic Acid B	FA-RTI mice	↓tubular injury	↓ ERS	Mai, et al. (2020)
Berberine	UUO rats	↓ECM, inflammation, OX	↓TGF-b1/Smad3	Wang, et al. (2014)
	DKD Murine	improve metabolism; ↓podocyte damage, glomerulosclerosis, mitochondrial dysfunction	↓mitochondrial ROS	Qin, et al. (2020)
			↑PGC-1α	
	STZ-DN	↓proteinuria, TIF, podocytes injury	↑AMPK phosphorylation	Sun et al. (2015); Zhang et al. (2020b)
			↓NF-k-light-chain-enhancer, TGFb1/Smad3	Li and Zhang, (2017)
			↑Drp1	Qin, et al. (2019)
	STZ-DN	↓inflammation	↓TLR4/NF-kB	Sun et al. (2015)
				Zhu et al. (2018)
	STZ-DN	↓ECM	↓Nrf2; regulating MMPs/TIMPs	Ni, et al. (2015)
	STZ-DN	↑renal pathology	↑GRKs	Wang, et al. (2013)
	SHR	↓hypertension, renal damage	↓RAS, IL-6, IL-17, IL-23	Guo, et al. (2015b)
	2K1C-RV-HTN rats	↓hypertension, sympathoexcitation	ROS/Erk1/2/iNOS	Tian, et al. (2019)
tripterygium glycosides	NUE-STZ-DN	↓glomerulosclerosis, TIF, microinflammation	↓macrophage infiltration, TNF-a, IL-1b, TGF-b1, p38 MAPK, NF-kB	Wu, et al. (2017)
Triptolide	STZ-HFD-DN	↓MA, inflammation, pathological damage	regulating Th cell balance	Guo et al. (2016)
			↓macrophage infiltration	
	STZ-HFD-DN	↓renal EMT	MiR-188-5p-PI3K/AKT	Xue et al. (2018)
	STZ-DN	↓renal ECM	↓microRNA-137/Notch1	Han, et al. (2018)
	STZ-DN	restoring autophagy	↓miR-141-3p/PTEN/Akt/mTOR	Li, et al. (2017b)
	UUO rats	↓inflammatory, ECM; immune activity	↓TGF-β1, CTGF, MCP1, osteopontin	Yuan, et al. (2011)
	PKD adult rats	↓disease progression; ↑renal function	↓JAK2-STAT3	Jing, et al. (2018)
	DOCA-salt hypertension	↓pathological damage	↓inflammatory	Zhang, et al. (2020a)
	FSGS rats	↓kidney injury, podocyte apoptosis	↓ IL4	Li, et al. (2020c)
Puerarin	UUO murine	↓ECM, TIF, epithelial cell apoptosis	↓NOX4; ↓phosphorylation of p38, ERK, JNK, MAPK	Zhou, et al. (2017)
	STZ-DM eNOS(−/−) mice	↓OX, albuminuria, kidney injury	↓NOX4↑; deacetylation of SIRT1-NF-κB	Li et al. (2017a); Xu, et al. (2020c)
	STZ-DN	↓pathological damage, apoptosis	↑miRNA-145-5p	Li et al. (2017a); Xu, et al. (2020c)
			↓TLR4/MyD88/NF-κB (p65)	
	STZ- DN mice	↓UACR, kidney injury	↑HMOX1, Sirt1-mediated podocyte autophagy	Li, et al. (2020b)
	STZ- DN rats	↓kidney hypertrophy, OX, podocyte injury	↑nephrin, podocin	Zhong, et al. (2014)
			↓MMP9	
	STZ-DN mice	↑autophagy, nephrin, podocin, podocalyxin	↑ PERK/eIF2α/ATF4	Xu et al. (2020b)
			↓HIF-1alpha, VEGF	Shukla, et al. (2017)
	STZ-DN rats	↓renal AGEs contents	↓AGEs, RAGE	Shen, et al. (2009)

↑: increase or activation or improve; ↓: decrease or inhibition; AAI: aristolochic acid I; ADR: Adriamycin; ADRN: adriamycin nephropathy; AD-PO-UAN: adenine and potassium oxonate-induced uric acid nephropathy mice; AMPK: AMP-activated protein kinase; BMP-7: bone morphogenetic protein 7; CTGF: connective tissue growth factor; CIN: Contrast-Induced Nephropathy; DN: diabetic nephropathy; DM: diabetes mellitus; DKD: diabetic kidney disease; Drp1: dynamin-related protein 1; ECM: extracellular matrix; EMT: epithelial-mesenchymal transition; EMTD: epithelial-myofibroblast *trans*-differentiation; ERS: endoplasmic reticulum stress; eNOS(−/−): endothelial nitric oxide synthase-null mice; FA-RTI: fatty acids-induced renal tubular injury; FSGS: focal segmental glomerular sclerosis; GRKs: G protein-coupled receptor kinases; HFD: high-fat diet; KI: kidney injury; MMPs: matrix metalloproteinases; MCD: minimal change disease; NF: nuclear factor; NLRP3: NLR Family Pyrin Domain Containing 3; OX: oxidative stress; PGC-1: peroxisome proliferator-activated receptor-gamma coactivator-1; PKD: polycystic kidney disease; PO: potassium oxonate; PPAR: peroxisome proliferator-activated receptor; RIRI: renal ischemia-reperfusion injury; SHR: spontaneously hypertensive rats; STZ: Streptozotocin; STZ-DN: streptozotocin induced diabetic nephropathy; STZ-HFD-DN: streptozotocin induced and high-fat diet diabetic nephropathy; UACR: urinary albumin creatinine ratio; MA: urine micro-albumin; UNE: unilateral nephrectomy; UPPR: rat models were induced by uninephrectomy, potassium oxonate, and proinflammatory diet; UUO: unilateral ureteral obstruction; TIF: tubulointerstitial fibrosis; TIMPs: tissue inhibitor of metalloproteinases; TNF-a: tumor necrosis factor-a; TGF-b1: transforming growth factor-b1; 2K1C-RV-HTN:Two-kidney, one-clip renovascular hypertensive rats.

**TABLE 2:** Clinical studies on the efficacy of CHM in the CKD.

Chinese herbal name	Disease	N	Therapeutic arms	Primary outcome	Study period	References
Abelmoschus Manihot (Huangkui capsule)	IgAN (24hUTP 0.5–3.0 g/d, eGFR≥ 45 ml/min/1.73 m^2^)	1,470	Huangkui capsule/placebo vs. losartan placebo	24 h UTP (mg/d)	48 weeks	Li, et al. (2020a)
	DN	5,895	Huangkui capsule + RAS blocker vs. RAS blocker	24 hUTP (g/d); UAER (mug/min); SCr(umol/L)	Meta-Analysis	Shi, et al. (2019)
	CKD1-2, primary glomerular disease (biopsy), moderate proteinuria	417	Huangkui capsule vs. losartan vs. Huangkui capsule + losartan	24 hUTP (mg/d)	24 weeks	Zhang, et al. (2014)
Tanshinone	CKD	1,857	Tanshinone vs. control	24 hUTP (g/d); eGFR	Meta-Analysis	Zhou, et al. (2020)
Tanshinone IIA	hypertensive nephropathy	1,696	Tanshinone IIA/ARBs vs. ARBs	eGFR	Meta-Analysis	Xu, et al. (2019)
Berberine	hypertensive patients with type 2 diabetes mellitus	69	control vs. berberine add-on	UACR (µg/mg)	2 years	Dai, et al. (2015)
Tripterygium Glycosides	DN	1,810	tripterygium glycosides + ARBs vs. ARBs	24 hUTP (g/d); UAER (mg/min); SCr	Meta-Analysis	Wu, et al. (2020a)
Tripterygium Wilfordii	DKD (stage IV)	1,414	Tripterygium Wilfordii +(ARB/ACEI) vs. (ARB/ACEI)	24 hUTP (g/d); Alb(g/l); TER	Meta-Analysis	Ren, et al. (2019)
Tripterygium Preparations	CKD	4,386	Tripterygium preparations vs. placebo, standard care, or other immunosuppressive treatment	UPE; SCr(mg/dL); CR; PR; relapse	Meta-Analysis	Zhu, et al. (2013)
Tripterygium glycosides	DN	70	Tripterygium glycosides + ARBs vs. ARBs	24 h UTP	48 weeks	Lengnan, et al. (2020)
Puerarin	DN(Stage III)	669	Puerarin + ACEI vs. ACEI	UACR (mug/min)	Meta-Analysis	Wang, et al. (2015a)

24 h UTP: 24 h urinary total protein; Alb: serum albumin; ACEI: angiotensin-converting enzyme inhibitors; ARB: angiotensin receptor blockers; CKD: chronic kidney disease; CR: complete remission; DN: diabetic nephropathy; DKD: diabetic kidney disease; eGFR: estimated glomerularfiltrationrate; IgAN: IgA nephropathy; PR partial remission; RAS: renin angiotensin system; SCr: serum creatinine; TER: total effective rate; UACR: Urinary Albumin Creatinine Ratio; UAER: urinary albumin excretion rate; Tripterygium Preparations: Tripterygium glycoside tablets, Tripterygium hypoglaucum Hutch tablets, and Tripterygium granules or extracts.

## *Abelmoschus manihot* and Huangkui capsule

*Abelmoschus manihot*, also called as “*Huangkui*” in Chinese, is an annual flowering herb plant in the family of Malvaceae. As a traditional Chinese medicine (TCM), the ethanol extract of the flower in *Abelmoschus manihot* is made as *Huangkui* capsule and has been used for medication of the patients with kidney diseases. Studies have confirmed that the major pharmacologically bioactive constituents in the flower of Abelmoschus Manihot are seven flavonoids, including Rutin, Hyperoside, Hibifolin, Isoquercetin, Myricetin, Quercetin, and Quercetin-3-O-robinobioside (Guo, et al., 2015a).

### Animal Studies

The biological effects of *Abelmoschus manihot* have been studied in several animal models of CKD, including 5/6 nephrectomy (Gu, et al., 2020), adriamycin-induced nephropathy (Li, et al., 2019), and streptozotocin-induced diabetic nephropathy (DN) (Liao, et al., 2019). The overall results show that treatment with *Abelmoschus Manihot* can improve kidney function, attenuate kidney damage and tubulointerstitial fibrosis, and reduce proteinuria (Cai, et al., 2017). These beneficial effects are related to inhibition of inflammation (Li, et al., 2019), anti-oxidative stress (Liao, et al., 2019), inhibiting renal epithelial-mesenchymal transition (EMT) (Gu, et al., 2020; Peng, et al., 2016), remodeling the intestinal microbiota and inhibiting micro-inflammation (Tu, et al., 2020). Mechanistically, Abelmoschus Manihot is able to suppress ROS-ERK1/2-mediated NLRP3 (NLR Family Pyrin Domain Containing 3) inflammasome activation (Li, et al., 2019), reduce tumor necrosis factor-α (TNF-α) and transforming growth factor-β1 (TGF-β1) protein expression (Tu, et al., 2013), inhibiting p38MAPK signaling pathway (Tu, et al., 2013) and autophagy-mediated macrophage polarization (Tu, et al., 2020). *Abelmoschus manihot* can also prevent glomerular podocyte apoptosis (Zhou, et al., 2012) by a mechanism associated with activating peroxisome proliferator-activated receptor (PPAR)-alpha/gamma (Ge, et al., 2016), inhibiting iRhom2/TACE signaling pathway (Liu, et al., 2017), attenuating endoplasmic reticulum stress (ERS) (Ge, et al., 2016; Liu, et al., 2017), and regulating autophagy and mitochondrial dynamics (Kim, et al., 2018). Moreover, *Abelmoschus manihot* has an ability to reduce oxidative stress and inflammation via modulation of AMPK (AMP-activated protein kinase)-Sirt1-PGC-1α (peroxisome proliferator-activated receptor-gamma coactivator-α) signaling axis (Liao, et al., 2019) and NADPH oxidase/ROS/ERK pathway (Cai, et al., 2017).

### Clinical Studies

*Abelmoschus manihot* is one of the important drugs for the treatment of CKD. It has been reported that treatment with *Abelmoschus manihot* can reduce proteinuria and improve renal function in patients with diabetic kidney disease (DKD) (Shi, et al., 2019), IgA nephropathy (Li, et al., 2020a), and CKD stages 1–2 (Zhang, et al., 2014). In a meta-analysis identified 72 clinical investigations involving 5,895 participants. Compared to a RAS blocker alone, combined treatment of Abelmoschus Manihot with a RAS blocker was more effective in reducing 24 h urinary protein (24 h UP), urinary albumin excretion rate (UAER), and serum creatinine (SCr) levels. *Abelmoschus manihot* did not increase adverse events (Shi, et al., 2019). Recently, a multicenter randomized controlled clinical trial for determining the efficacy of Abelmoschus Manihot were conducted in a total of 417 patients with biopsy-proven primary glomerular disease (Zhang, et al., 2014) (CKD stage 1–2, with moderate proteinuria) from 26 hospitals in China. The results show that *Abelmoschus manihot* can effectively reduce urine protein, and no obvious adverse events were identified. In another multicenter randomized clinical trial, 1470 biopsy-proven IgAN patients (proteinuria between 0.5 and 3.0 g/d and eGFR of >/ = 45 ml/min/1.73 m^2^ were treated with either *Abelmoschus manihot* or losartan at 1:1, observed for 48 weeks. The results indicated that the effectiveness of *Abelmoschus manihot* was similar to losartan in reducing urinary proteins (Li, et al., 2020a). The eGFR was stable and did not show a significant decline in both treatment groups during the 48-weeks follow-up. The rates of adverse events did not differ between the two treatment groups (Li, et al., 2020a). Thus, *Abelmoschus manihot* can be used to treat patients who may not tolerate ACEI/ARB due to hypotension or other disease. As such, *Abelmoschus manihot* appears effective and safe in improving proteinuria and preserve renal function in patients with CKD. The long-term benefits of *Abelmoschus manihot* in reducing the risk of progressive renal dysfunction remain unclear and need further study.

## *Salvia miltiorrhiza* and Its Components: Tanshinone and Salvianolic Acid

*Salvia miltiorrhiza*, also called “*Danshen*” in Chinese, is a popular Chinese herb from dried roots of *S. miltiorrhiza* Bunge, has been used for over 2,000 years for the treatment of cardiovascular diseases without obvious side effects (Yan, et al., 2018). The active ingredient of *Danshen* is tanshinone, which contains more than 50 compounds such as tanshinone I, tanshinone IIA, and tanshinone IIB as well as the water-soluble compounds salvianolic acid A (SAA), salvianolic acid B, and tanshinol (Xue, et al., 2019). Injection of Sodium tanshinone IIA sulfonate (STS), the extract of *Danshen*, has been widely used in current clinical practice in China to treat CKD in recent years.

### Animal Studies

Several models of CKD were used to study the efficacy and action of mechanisms of *Salvia miltiorrhiza.* In the five-sixth nephrectomy model (Zhang, et al., 2019b) (Zhang, et al., 2019a) (Zhang, et al., 2018) (Wang, et al., 2015b), administration of Salvianolic acid A (SAA) and Tanshinone IIA were shown to attenuate oxidative stress and inflammation by activating the Akt/GSK-3beta/Nrf2 signaling pathway and up-regulation of bone morphogenetic protein 7 (BMP-7) and Smad6 as well as inhibiting NF-κB and p38 MAPK and TGF-β/Smad signaling pathways. In adriamycin (ADR)-induced minimal change disease (MCD) rat model, SAA exhibited a significant anti-proteinuria effect (Wang, et al., 2019b). In a rat model of STZ-induced diabetes, Tanshinone IIA and SAA attenuates renal damage via inhibiting oxidative stress and inflammation (Chen, et al., 2017) (Hou, et al., 2017). SAA restored glomerular endothelial permeability *via* AGE-RAGE-RhoA/ROCK and disturbed autophagy *via* AGE-RAGE-Nox4 axis (Hou, et al., 2017). Tanshinone IIA also reduced endoplasmic reticulum stress via attenuated PERK signaling activities (Xu, et al., 2020a). In UUO rats, Salvianolic acid B attenuates renal interstitial fibrosis by regulating the heparanase/syndecan-1 axis (Hu, et al., 2020), Salvianolic acid A and C reduced the secretion of renal inflammatory cytokines CCL5 and CXCL10 to protect renal function, improve tubular function and renal pathology (Li, et al., 2015). In adenine and potassium oxonate-induced uric acid nephropathy mice or renal ischemia-reperfusion injury rats, Tanshinone IIA suppressed oxidative stress-activated MAPK pathways (Zhang, et al., 2020c), and salvianolic Acid B modulates caspase-1-mediated pyroptosis *via* blocking Nrf2 Pathway (Pang, et al., 2020), suppressing oxidative stress and inflammation through activation of PI3K/Akt signaling pathway (Ma, et al., 2017b). Furthermore, tanshinone IIA attenuates contrast-induced nephropathy *via* enhancing Nrf2/ARE activation rats (Liang, et al., 2018). Tanshinone I protects mice from aristolochic acid I-induced kidney injury by induction of cytochrome P450 1A (Feng, et al., 2013). Salvianolic Acid B protects against fatty acid-induced renal tubular injury *via* inhibition of endoplasmic reticulum stress, play an important role in obesity-related kidney injury (Mai, et al., 2020). Overall, Salvia miltiorrhiza can protect against kidney disease through diverse mechanisms involved in inhibiting multiple profibrotic pathways.

### Clinical Studies

Many small-clinical trials have been performed to evaluate the efficacy of *Salvia miltiorrhiza* in patients with CKD. A recent meta-analysis has summarized the results of *Salvia miltiorrhiza* (tanshinone) for CKD treatment (Zhou, et al., 2020). Twenty-one studies were reviewed in this meta-analysis, which involved 1857 patients including 954 cases from the *salvia miltiorrhiza* treatment group and 903 cases from the control group. It was found that *Salvia miltiorrhiza* could reduce urine protein levels, improve kidney function, and attenuate CKD without significant side effects. Among 21 studies included in this meta-analysis, only 2 evaluated the safety of tanshinone, tanshinone administration did not significantly the side effects.

Recently, another meta-analysis assessed the efficacy and safety of Sodium tanshinone IIA sulfonate in treatment of hypertensive nephropathy. Sixteen clinical trials involving 1,696 patients were included in this meta-analysis. It was interestingly found that a combination of Tanshinone IIA (TIIA) and angiotensin receptor blockers (ARBs) was more effective than ARB monotherapy in modulating hypertensive nephropathy (Xu, et al., 2019). This was indicated by improved eGFR and reduced urinary protein, serum creatinine, cystatin-C, and better control in systolic blood pressure (SBP) and diastolic blood pressure (DBP) in group combined with STS plus ARBs than in ARBs alone group. Thus, it appears that STS can be used as an adjuvant agent in the management of hypertensive nephropathy. Nevertheless, all included trials in this meta-analysis report were published in Chinese, sample size for individual trials were small and the treatment course was short (2–4 weeks). To achieve more conclusive results, other large-scale, multicenter, long-term and rigorously designed RCTs should be conducted in the future.

## *Rhizoma coptidis* and Its Components Berberine

*Rhizoma coptidis* is known as “*Huanglian*” in Chinese. Modern pharmacological studies have demonstrated that *Rhizoma coptidis* and its component berberine have various pharmacological activities, including anti-inflammatory, hypoglycemic, antihypertensive, antibacterial, and other effects.

### Animal Studies

The potential effects of berberine on renal interstitial fibrosis has been examined in animal models of UUO, DKD and hypertensive nephropathy. In a rat model of UUO, it was found that administration of berberine (200 mg/kg per day) attenuated deposition of extracellular matrix, inhibited inflammation, reduced oxidative stress and suppressed TGFβ1/Smad3 signaling pathways (Wang, et al., 2014). In a murine model of DKD, berberine treatment was observed to reverse the disordered metabolism, podocyte damage, glomerulosclerosis, lipid accumulation, excessive generation of mitochondrial ROS, mitochondrial dysfunction, and deficient fatty acid oxidation through a mechanism associates with inactivation of the PGC-1alpha signaling pathway (Qin, et al., 2020). Berberine could also significantly inhibited urine protein excretion, ameliorated tubulointerstitial fibrosis, protect glomerular podocytes in this model (Sun, et al., 2015, Zhang, et al., 2020b, Li and Zhang 2017, Qin, et al., 2019). The renoprotective effect of berberine is related to activating autophagy *via* AMPK phosphorylation (Zhang, et al., 2020b), inhibiting inflammation *via* suppressing TLR4/NF-κB pathway (Sun, et al., 2015) (Zhu, et al., 2018), inhibiting the Nrf2 pathway, and regulating the proteins expression of the matrix metalloproteinases (MMPs)/tissue inhibitors of metalloproteinases (TIMPs) (Ni, et al., 2015) and G protein-coupled receptor kinases (GRKs) (Wang, et al., 2013). In addition, berberine could delay the onset and attenuate the severity of hypertension, ameliorate hypertension-induced renal damage in spontaneously hypertensive rats. This action of berberine is associated with inhibition RAS activities and expression of some pre-inflammatory cytokines IL-6, IL-17, and IL-23 (Guo, et al., 2015b). The anti-hypertension and anti-inflammatory effects were also observed in two-kidney, one-clip (2K1C) renovascular hypertensive rats, associated with inhibition of the ROS/Erk1/2/iNOS pathway (Tian, et al., 2019).

### Clinical Studies

Clinical studies have shown that berberine treatment is beneficial for hypertensive patients with T2DM (Dai, et al., 2015). In a 2-years random clinical trial on 69 hypertensive patients with T2DM, whose blood pressure and fasting plasma glucose were adequately controlled prior to the study, were enrolled and randomly assigned into add-on (36 cases) and control (33 cases) groups. Berberine was orally administrated to the patients in the add-on group concomitantly with standard hypotensive and hypoglycemic treatment. Adequately control of blood pressure and glucose was observed in those two groups. In the berberine add-on group of patients, a significant reduction in UACR, urinary osteopontin and KIM-1 was further observed in addition to improved renal hemodynamics, reduced renal inflammation, and oxidative stress. Therefore, berberine might be used as an alternative therapeutic strategy for the management of kidney injury. Other clinical trials are needed to investigate the efficacy of berberine in CKD induced by other etiologies.

## *Tripterygium wilfordii* and Its Components: Triptolide, Tripterygium Glycosides

Triptolide and tripterygium glycosides are the main bioactive constituents isolated from the Chinese herb *Tripterygium wilfordii*, also called as “Leigongteng.” Pharmacological studies have shown that Triptolide exhibits multiple effects, including renal protective, antitumor, anti-inflammatory, immunosuppressive, cardioprotective, antiangiogenesis activities, and multiorgan toxicity effects. Triptolides have been extensively used to treat some primary and secondary kidney disease such as nephritis, minimal change disease and membranous nephropathy in humans. Its anti-fibrotic effects have been also reported in some of animal models as indicated below.

### Animal Studies

In a rat model of diabetic nephropathy, tripterygium glycosides was shown to attenuate glomerulosclerosis and interstitial fibrosis and exert anti-microinflammatory effects (Wu, et al., 2017). The effect of Triptolide-elicited renoprotection in diabetic nephropathy is associated with regulating T cell balance and reducing macrophage infiltration to the kidney (Guo, et al., 2016), attenuates renal tubular EMT (Xue, et al., 2018), prevents extracellular matrix accumulation by targeting microRNA-137/Notch1 (Han, et al., 2018) and miR-141-3p/PTEN/Akt/mTOR pathway (Li, et al., 2017b)**.** In a rat UUO model, treatment with Triptolide also decreased interstitial collagen deposition, inhibited renal interstitial fibroblast activation and suppressed production of proinflammtory and profibrogenetic factors, including TGF-β1, connective tissue growth factor (CTGF), MCP1 and osteopontin (Yuan, et al., 2011). Additionally, triptolide administration significantly delayed disease progression and improved renal function in an adult rat model of polycystic kidney disease through inhibiting the JAK2-STAT3 pathway (Jing, et al., 2018), attenuates renal damage by limiting inflammatory responses in DOCA-salt hypertension (Zhang, et al., 2020a), and inhibits podocyte apoptosis by targeting IL4 to alleviate kidney injury in FSGS rats (Li, et al., 2020c).

### Clinical Studies

Two meta-analyses (Ren, et al., 2019; Wu, et al., 2020a) evaluated the clinical efficacy and safety of Tripterygium wilfordii/tripterygium glycosides combined with ARB/ACEI in the treatment of stage IV DKD. In the controlled trial (RCT), 1414 participants (Ren, et al., 2019) were evaluated in detail, and another meta-analysis included 23 studies, including a total of 1810 DN patients (Wu, et al., 2020a). Tripterygium wilfordii/tripterygium glycosides combined with ARB/ACEI significantly improved 24-h urinary total protein (24 h-UTP), urinary UAER, SCr, and albumin more than did ARB/ARB alone. Some minor side effects such as abnormal liver function tests were observed in the combined treatment group, with the risk of adverse reactions increased by 8%. Moreover, a prospective, randomized controlled trial for assessing the efficacy of tripterygium wilfordii in Stage IV-DN is still in progress (Lengnan, et al., 2020).

A systematic review and meta-analysis of Tripterygium wilfordii polyglycosides in the treatment of CKD indicate that among 75 trials that included 4,386 participants, treatment with tripterygium polyglycoside preparation reduces proteinuria, lowers serum creatinine, improves the complete remission rate by 56%, improves the complete or partial remission rate by 24%, and reduces the relapse rate by 58% (Zhu, et al., 2013). Tripterygium polyglycoside preparation group also increased liver function abnormalities and menstrual changes (Zhu, et al., 2013).

## Kudzu Root Pueraria and Its Component Puerarin

Puerarin is natural flavonoid extracted from the Chinese medical herb Radix puerariae, also called as “*Gegen*”. Many studies have demonstrated that puerarin has a renoprotective effect in animal model of AKI induced by various nephrotoxicants such as cisplatin (Wu, et al., 2020b) (Ma, et al., 2017a), methotrexate (Liu, et al., 2018), lead (Liu, et al., 2012) and carbon tetrachloride. Recently, the effect of puerarin on CKD and mechanism involved have also been examined in animal models of UUO and DA.

### Animal Studies

Puerarin treatment attenuates renal tubulointerstitial fibrosis in a murine model of UUO, as evidenced by decreased the accumulation of ECM and reduced renal tubule damage. Mechanistically, puerarin inhibited renal epithelial cell apoptosis, reduced expression of NOX4 and inhibited phosphorylation of phosphorylation of p38, ERK, and JNK, three MAPK pathways associated with renal fibrosis (Zhou, et al., 2017). In a murine model of diabetic nephropathy, puerarin also exhibits a potent renoprotective and anti-fibrotic effect through a mechanism associated with suppression of NOX4 and miRNA-140-5p expression (Li, et al., 2017a; Xu, et al., 2020c), promotion of podocyte autophagy (Li, et al., 2020b), down-regulation of MMP9 (Zhong, et al., 2014), and activation of the PERK/eIF2α/ATF4 signaling pathway (Xu, et al., 2020b). In addition, puerarin also reduces the contents and expression of advanced glycation end products in the diabetic kidney (Shen, et al., 2009) and restores the expression of nephrin by inhibiting the expression of HIF-1alpha and VEGF (Shukla, et al., 2017).

### Clinical Studies

A meta-analysis has assessed the beneficial and harmful effects of puerarin plus ACEI compared with ACEI alone for the treatment of individuals with stage III DN. Ten RCTs involving 669 participants were included in this meta-analysis. All trials were conducted in China and published in Chinese. Treatment of DN with puerarin plus ACEI significantly decreased the urinary albumin excretion rate (UAER) but had no effect on 24-hUTP. One trial reported abdominal discomfort and nausea (2 cases) in the treatment group. Although these studies suggest that puerarin can reduce proteinuria of individuals with stage III DN (Wang, et al., 2015a). Further clinical trials with more samples and multiple centers should be conducted to verify the beneficial results for DN.

## Common Mechanisms of Chinese Herbal Medicines in Treating Kidney Disease

**FIGURE 1:**
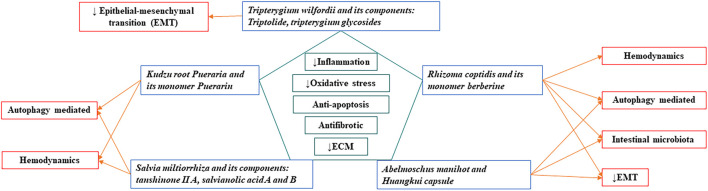
Common mechanisms of Chinese herbal medicines in treating kidney disease.

Combining the content of this article and previous literature review (Zhao, et al., 2020; Zhong, et al., 2013), we identify that eight Chinese herbal medicine have anti-inflammatory, anti-oxidant, anti-apoptotic effects, reducing extracellular matrix deposition, and anti-fibrosis. Among them, *Salvia miltiorrhiza, Rhizoma coptidis, Abelmoschus manihot* can interrupt almost all the processes leading to renal fibrogenesis. A summary of the common mechanisms of these Chinese herbal medicines in the treatment of kidney diseases is shown in Figure 1.

## Conclusion and Perspective

Although preclinical animal studies often indicate therapeutic benefits of Chinese herbal medicine in models of CKD, convincing evidence for or against Chinese herbal medicine for patients with CKD is limited. Here we summerized recent advances on the therapeutic effect of five Chinese herbal compounds, either single-herbal or monomer, on CKD. Their anti-fibrotic effects are involved in the regulation of immunity, alteration of hemodynamic changes, anti-oxidative stress and fibrosis. Clearly, more research is needed to identify the active ingredients of herbal medicines effective for the treatment of CKD and the mechanism of action involved. Determination of a monomer with definite curative effect and mechanism of action, and optimization of the formulation of TCM through modern scientific research will further improve and confirm the clinical curative effect. Below are several issues that should be considered.

First, clinical efficacy research should be strengthened. Although some single Chinese medicines or monomers have conducted RCT studies and meta-analysis, such as “Huangkui” capsules, most others have only small samples of clinical controlled observations, such as berberine. Therefore, a large-sample, long-period RCT studies should be initiated on the basis of physicians’ personal clinical experience and small-sample clinical observations to confirm the clinical efficacy of TCM against renal fibrosis.

Second, mechanism study should be focused on the most critical action of mechanism in a given drug. For example, tanshinone IIA, berberine, and triptolide all have anti-inflammatory and anti-oxidant actions, but it remain unclear which herb is stronger in those actions. Puerarin has anti-inflammatory and antioxidant effects, regulating podocyte autophagy, tubular epithelial cell autophagy and apoptosis. It is unknown which is the most critical action of puerarin against renal fibrosis. A recent study (Zhong, et al., 2019) showing that arctigenin attenuates diabetic kidney disease through the activation of PP2A in podocytes gives us a good example and enlightenment to pursue in depth understanding of modern pharmacology of Chinese herbal medicine.

Third, the role and action of mechanism of monomer compounds need to be explored in depth**.** The research on the combination of Chinese medicine monomers is worth of exploration in the future. *Salvia miltiorrhiza* and *Rhizoma coptidis* often appear in one prescription of Chinese herbal medicine to treat CKD such as Shenshuaining Capsule (Cui, et al., 2016), Yishen Zhishuai Granules and Shenshuai II Recipe (Wang, et al., 2019a). According to the classic theory of Chinese traditional Medicine, *Rhizoma coptidis* is capable of clearing heat and dampness, purging fire and detoxification, as well as inducing Qi, whereas *Salvia miltiorrhiza* has an ability to activate blood and remove stasis as well as cool blood and detoxify. The combination of these two herbs can act in concert to eliminate the most common pathogenic syndromes of CKD, such as dampness, heat, blood stasis, and toxicity. Future research is necessary to explore the synergistic effect and mechanism of berberine and salvianolic acid on renal fibrosis in order to further elucidate the clinical significance and mechanism of action of *Salvia miltiorrhiza* and *Rhizoma coptidis*.

Fourth*,* the systems approach is necessary for exploring the synergistic effects of TCM in kidney disease. The systems approach in TCM is a methodology that combines computational and experimental tools to discover novel therapeutic agents, identify their candidate targets, and understand their therapeutic mechanisms. Traditional medicine often is a mixed formula composing of several types of herbs, and a formula with mixed herbs usually works better than single ones. However, the mechanism of action of individual herbs and their synergistic effects in a formula are frequently unknown, thus, the systems approach should be utilized to analyze the interactions among compounds in order to determine the synergistic effects of TCM in a given formula. To achieve this goal, we need to 1) identify the active ingredients from traditional medicine mixtures using modern technologies such as gas chromatography-mass spectrometr, 2) identify targets of active ingredients by developing predictive network models and analyzing the complex interactions among herbs, compounds, herb-target, and compound-target networks. 3) determine the biological activity and toxicity profile of individual active compounds and multiple ingredients in diverse combinations using both *in vitro* techniques and animal models of kidney disease, 4) elucidate the action of mechanism of each active compounds and their synergistic effects by using advanced systems approaches such as mass spectrometry and affymetrix microarrays for gene expression analysis, and 5) conduct clinical trial to assess the therapeutic effect of single active compounds and multiple ingredients with different combinations.
